# Phase coherence between precipitation in South America and Rossby waves

**DOI:** 10.1126/sciadv.aau3191

**Published:** 2018-12-19

**Authors:** Maximilian Gelbrecht, Niklas Boers, Jürgen Kurths

**Affiliations:** 1 Potsdam Institute for Climate Impact Research, Potsdam, Germany.; 2 Department of Physics, Humboldt University Berlin, Germany.; 3 Grantham Institute, Imperial College London, UK.; 4 Institute of Applied Physics of RAS, Nizhny Novgorod, Russia.

## Abstract

The dominant mode of intraseasonal precipitation variability during the South American monsoon is the so-called precipitation dipole between the South Atlantic convergence zone (SACZ) and southeastern South America (SESA). It affects highly populated areas that are of substantial importance for the regional food supplies. Previous studies using principal components analysis or complex networks were able to describe and characterize this variability pattern, but crucial questions regarding the responsible physical mechanism remain open. Here, we use phase synchronization techniques to study the relation between precipitation in the SACZ and SESA on the one hand and southern hemisphere Rossby wave trains on the other hand. In combination with a conceptual model, this approach demonstrates that the dipolar precipitation pattern is caused by the southern hemisphere Rossby waves. Our results thus show that Rossby waves are the main driver of the monsoon season variability in South America, a finding that has important implications for synoptic-scale weather forecasts.

## Teaser

Rainfall in South America follows a dipole-like pattern that is driven by atmospheric waves associated with the jet stream.

## INTRODUCTION

**Fig. 1:**
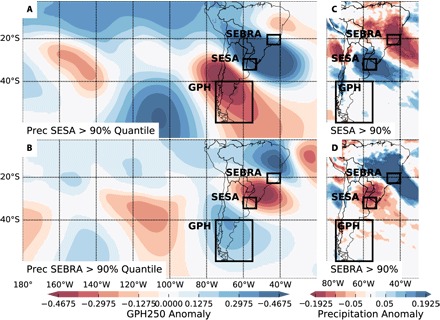
Precipitation regimes in austral summer in South America. Geopotential height (GPH) at 250 hPa and precipitation anomalies [with respect to the NDJF (November to February) climatology] for times when (**A**) and (**C**) precipitation in SESA is above its 90% percentile and (**B**) and (**D**) when precipitation in SEBRA is above its 90% percentile. For the calculation of the percentiles, only time steps with precipitation larger than 0.01 mm/day are considered. The reference regions SESA and SEBRA serve as a proxy for the South American precipitation dipole in this study, and the GPH in southern South America serves as a proxy for Rossby wave activity. The size and position of the GPH reference region are chosen, such that it roughly covers one-half of the spatial wavelength.

The South American monsoon system (SAMS) during the austral summer season is established by a pronounced low-level moisture inflow from the tropical Atlantic Ocean toward the South American tropics. After crossing the Amazon basin, this easterly flow is blocked by the Andes mountain range and subsequently channeled southward, providing the moisture for monsoonal precipitation in the subtropics (*1*–*3*). There exists considerable variability in the direction of this subsequent moisture flow to the subtropics, and related to this, precipitation in South America during the monsoon season exhibits substantial intraseasonal variability. The most pronounced variability mode is typically described as a precipitation dipole (*3*–*6*), with the strongest amplitudes of this alternating pattern found between Southeastern Brazil (SEBRA) and southeastern South America (SESA) (see Fig. 1). These two regions are also the northernmost and southernmost exit regions of the low-level flow, respectively. SEBRA is usually part of the climatological position of the South Atlantic convergence zone (SACZ) (*6*–*8*), one of the key characteristics of the SAMS. These two regions that are most affected by this variability pattern are among the most densely populated and agriculturally important areas in South America.

Previous research suggested that this mode of precipitation variability is related to the Madden-Julian Oscillation (MJO) and the Bolivian high (*1*, *3*, *6*, *9*). In addition, evidence has also been reported that the dipole is related to Rossby wave trains emanating from the southern Pacific region and their relative phasing with the MJO (*6*, *10*, *11*). Here, we focus on this relation to the Rossby wave trains, which we represent by the geopotential height (GPH) at 250 hPa in southern South America.

The aim of this study is to have a detailed analysis of the relationship between the eastward propagating Rossby waves and the dominant modes of precipitation variability in South America during the monsoon season. Previous studies mainly used principal components analysis (PCA) and composite analyses to analyze the characteristics of the precipitation variability in South America (*4*, *5*, *10*). More recently, complex network approaches were able to complement these approaches (*12*). However, aside from studying composite anomalies during or before precipitation events or the network topology induced by the synchronization of these events, these methods are not suitable to further investigate the detailed atmospheric mechanisms behind the dipolar precipitation variability and its relationship to the Rossby wave trains. In particular, a direct statistical test of this relationship based on suitably identified time series has, to our knowledge, not been performed so far.

The influence of Rossby wave trains on extreme events in other regions has been studied with method such as the wave activity flux before [e.g., (*13*, *14*)], but here, we intend to shed further light on the mechanism behind the dipole pattern by directly investigating its dynamical properties in terms of its statistical relationship with the relevant atmospheric dynamics. This will be done, on the one hand, by using a conceptual model that explains the observed structure of empirical orthogonal functions (EOFs) and, on the other hand, by showing that the reconstructed phases of the relevant observables—i.e., precipitation in the dipole regions and upper-level GPH in southern South America representing the Rossby wave train—are coherent with each other. The latter approach relies on concepts that have first been explored to study dependencies of chaotic oscillators in nonlinear dynamical systems theory: If two such systems are brought into contact by a weak coupling, then first, the phases of their respective variables adjust and synchronize (*15*). This framework has been applied successfully to climate time series before, investigating the coherence between El Niño–Southern Oscillation (ENSO) and the Indian monsoon (*16*). Using the methodological concept of phase coherence will allow us, particularly, to establish statistical significance of the relationship between the dipolar precipitation pattern and the Rossby wave train.

Since we investigate an intraseasonal phenomenon with these methods, we first remove the annual cycle and unwanted high-frequency oscillations and noise by preprocessing the data using singular spectrum analysis (SSA) (*17*, *18*). Alternative methods to accomplish the spectral decomposition and filtering are also discussed. All methods used to derive the results presented in the next section are explained in detail in Materials and Methods further below.

## RESULTS

### Conceptual model

**Fig. 2:**
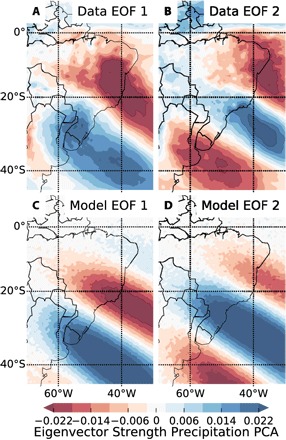
Data and model EOF analysis. (**A** and **B**) First and second EOFs of the precipitation data. NDJF precipitation anomalies were used to calculate these EOFs, which account for 9% of the total precipitation variability. A plot of the eigenvalue spectrum can be found in fig. S1. (**C** and **D**) First and second EOFs of the data generated by the conceptual model introduced in this article.

Typically, dominant modes of variability are identified and visualized on the basis of EOFs, which are obtained from a PCA of the covariance matrix (*19*). This approach, based on outgoing long-wave radiation data, also led to the first description of the South American precipitation dipole (*4*). The dipole pattern is recognizable in the two leading EOFs of the precipitation anomalies (top row of Fig. 2), which emphasizes the importance of this variability mode for South American climate.

It is possible to reproduce the spatial patterns of leading EOFs of the precipitation data in South America with a simple conceptual model, which corroborates the hypothesis that the precipitation dipole is caused by a propagating wave: The model accomplishes to explain the structure of the two leading EOFs of the precipitation anomalies by conceptually representing the Rossby wave train as a traveling wave of pressure anomalies *h*. In the conceptual model, this wave triggers precipitation *p* at the position where the wave’s gradient attains its maximum, in accordance with the basic mechanism of frontal systems

□h(x,t)=0 (1)

with the one-dimensional d’Alembertian $□=\frac{1}{c}\frac{\partial^{2}}{{\partial t}^{2}}-\frac{\partial^{2}}{{\partial x}^{2}}$ and *p* as its spatial derivative

$$p(x,t)=\frac{\partial }{\partial x}h(x,t)$$ (2)

which solves to a traveling wave along a propagation direction that is defined by an angle parameter θ. In addition, a Gaussian damping along and perpendicular to the propagation is added to localize the wave and roughly account for the orography. The parameters of the model equation (see Materials and Methods and the Supplementary Materials below for full details) are fitted via least squares to minimize the differences between the observation- and model-derived EOFs. Figure 2 shows the two leading EOFs of the observed precipitation anomaly data and the conceptual model. The qualitative structure of the EOFs is reproduced well by the conceptual model. The fitted value for the wavelength of the model wave is roughly 4000 km, while the alternating Rossby wave train pattern in Fig. 1 also exhibits a wavelength of about 4000 to 6000 km, measured as the distance between subsequent maxima. While small deviations from the EOFs of the precipitation data that could be caused by the orography or other external effects are to be expected with such a simple conceptual model, it can be inferred that the type of alternating EOF pattern that is present for the South American precipitation dipole can be caused by a propagating wave, such as a Rossby wave train, and the resulting pressure anomalies.

**Fig. 3:**
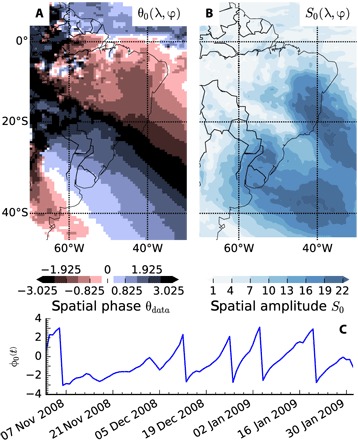
First CEOF of the NDJF precipitation anomalies. (**A**) Spatial phase θ_0_(λ, ϕ) and (**B**) spatial amplitude *S*_0_(λ, ϕ) of the first CEOF component. (**C** and **D**) Temporal phase and temporal amplitude of the 2008/2009 season. Only one season is shown to representatively show the qualitative behavior of these measures. The other seasons exhibit a similar behavior. (See Materials and Methods for a detailed account of CEOF analysis.)

This relationship becomes even clearer when, additionally, a complex EOF (CEOF) analysis is performed. CEOF analysis (see Materials and Methods for details) relies on performing a PCA on the time series augmented by its Hilbert transform as the imaginary part of the therefore complex time series, referred to as the analytical signal. The Hilbert transform is given by the convolution of the time series with 1/(π*t*) and induces a 90° phase shift of every Fourier component of the time series. This approach allows us to identify and analyze oscillatory behavior, as it adds information about a “future” state of the oscillation to the time series. Therefore, CEOFs allow us to assess oscillatory patterns and particularly patterns due to propagating waves, better than standard EOFs [e.g., (*20*, *21*)]. Figure 3 shows the spatial phase θ_0_(λ, ϕ) and amplitude *S*_0_(λ, ϕ) of the first CEOF—i.e., the dominant oscillatory pattern—in the top panel. For a propagating wave, one would expect a monotonously, constantly growing spatial phase along the propagation direction and constant values on lines perpendicular to this direction. This is exactly what the conceptual model exhibits (see fig. S2), and the data closely resample these as well. The spatial phase shows the propagation of a wave along the eastern coast of South America in a clear pattern extending from Argentina to the eastern tip of Brazil, with its spatial amplitude maxima on the continent close to the SESA and SEBRA reference regions. The temporal phase (Fig. 3C) exhibits a distinct oscillatory pattern as well: a seesaw pattern with roughly similar periods indicating the temporal dynamics of the dominant oscillation pattern. The CEOF thus also shows a southwest to northeast propagating oscillation pattern with its maxima close to SESA and SEBRA and temporal periods similar to those of Rossby wave trains.

### Phase coherence

#### Singular spectrum analysis

Complementarily to the conceptual approach, we perform a data-driven analysis of the dynamical properties of the precipitation dipole by investigating the phases of the three observables and their dependencies. We use SSA to remove the annual cycle and high-frequency noise from the time series (*17*, *18*). SSA can be briefly summarized as performing a PCA on the time-delay embedded time series. More details can be found in Materials and Methods. We note that Paegle *et al*. (*5*) had also used SSA to study specific frequency bands of variability related to the dipole pattern under study. According to the intraseasonal frequency range that we are interested in, the SSA is carried out with a delay τ = 60 days. The eigenspectrum of all investigated time series is shown in fig. S3. The SSA decomposes the signal into 60·4 components ordered by the magnitude of their eigenvalues, as there are four data points per day in the dataset. We consider three different approaches to identify the right components for our analysis and ultimately combined our knowledge from all three of them.

First of all, generating surrogates can provide us with significance thresholds for the eigenvalues; this is referred to as Monte Carlo SSA (MCSSA) (*22*). Here, we use 1000 shuffle surrogates. The eigenvalues of the first 25 components, and those of the corresponding surrogates, are shown in fig. S3. If the eigenvalue of the *k*th component is lower than that of the *k*th shuffle MCSSA surrogate, then the corresponding reconstructed components are regarded as noise. This yields similar results to the visual check of the cumulative eigenvalue series approaching a horizontal line (*23*). In our case, the cumulative explained variance of the eigenvalues, which is directly proportional to the cumulative eigenvalue series itself, is larger than 95% for all three observables at this point. As the eigenvalue spectrum differs for each of the observables, the MCSSA significance test does so as well. The break point is at *k* = 19 for SESA, *k* = 16 for SEBRA, and *k* = 25 for GPH.

Another way of approaching the problem to select the right components is to directly choose those component ranges that induce the smallest phase differences (the phase reconstruction is described in the next section). As we have three sets of components to choose from, this is a highly nontrivial optimization problem. We used a genetic algorithm (*24*) whose individuals are lists of the starts and ends of the component ranges of the three time series. The fitness used in this algorithm is the phase difference at the end of the series, modified with extra penalties to favor larger component ranges. This results in the ranges 4 to 15 for SEBRA, 3 to 13 for SESA, and 2 to 14 for GPH.

Last, it should be assured that the components that we choose actually exhibit oscillations within the intraseasonal frequency band that we are interested in. For this purpose, we calculate the dominant frequencies of all components. The first two reconstructed components of all three observables contain the annual cycle, and the third components exhibit dominant frequencies between 1/(40 days) and 1/(50 days). SESA’s and GPH’s fourth components are within this range as well. Reconstructed components with *k* > 12 exhibit frequencies *f*_dom_ > 1/(10 days), and components with *k* > 15 exhibit frequencies *f*_dom_ > 1/(8 days). Thus, the ranges suggested by the optimization routine described in the previous paragraph include only significant reconstructed components, and all reconstructed components with intraseasonal dominant frequencies except for those with frequencies around 1/(40 days). The latter frequency range is not typically associated with Rossby wave trains. However, we also tested to include these components and found qualitatively similar results, indicating that this approach is robust.

All further investigations are carried out with time series attained by summing the components found by the optimization. Since the data are linearly detrended before the SSA, and the annual cycle was removed via SSA, the time series oscillate around zero. To validate that the SSA-filtered time series still reflect the precipitation dipole, we check whether the extreme events in the two dipole reference regions (defined as the time points with precipitation above the 90th percentile of the unprocessed data) still exhibit positive values in the processed time series. This is the case for 95% of the SEBRA events and 97% of the SESA events. Since an extreme event-based definition was able to capture the characteristics of the precipitation dipole in a previous study (*12*), we are convinced that the SSA-filtered time series, which preserve these events and consist of most of the reconstructed components with dominant frequencies in the intraseasonal range, still represent the precipitation dipole.

The results on the coherence of phases presented below are robust for different approaches to preprocess the data: Alternatively to SSA, it would also be possible to process the data with a regular bandpass filter. However, one needs to carefully select a filter with constant phase response in the frequency range we are interested in, and one also needs a priori knowledge about the cutoff frequencies. We used a Lanczos 10- to 50-day bandpass filter with a high number of weights (*25*) and found qualitatively similar results: Phase differences at the end of the time series are slightly larger than for the optimization approach described above but still significantly smaller than those of the surrogates. Corresponding results can be found in figs. S5 to S8. The phase difference histograms shown below retain their form very closely. We additionally tested ensemble empirical mode decomposition (EEMD) (*26*) as an alternative, and similar results can be obtained; however, identifying the right intrinsic mode functions of the EEMD is more challenging.

#### Phase reconstruction

**Fig. 4:**
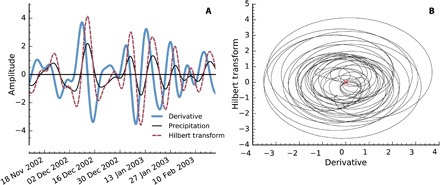
Phase embedding. (**A**) Example of a processed observable (SEBRA), its derivative, and the Hilbert transform of the derivative for the 2002/2003 NDJF season. (**B**) Example of the embedding of a processed observable (SEBRA) via Hilbert transform, for three consecutive seasons 2002/2003, 2003/2004, and 2004/2005.

From the nonlinear dynamical systems theory, we know that the phases of two coupled oscillatory systems will adjust and synchronize [e.g., (*15*)]. In the following, we show that the precipitation dipole and the Rossby wave train exhibit a significant phase coherence. The phases of the SSA-processed time series are reconstructed by embedding them with a Hilbert transform (see Materials and Methods). The seasonality of the data was taken into account by performing an end point matching between subsequent seasons. Figure 4 shows an example of the embedding, and that almost all oscillations revolve around the origin. This demonstrates that the phase can be defined meaningfully even across seasons, and the relation of the phase time series of the two precipitation proxies and the Rossby wave train proxy can be compared to each other.

#### Phase relation

**Fig. 5:**
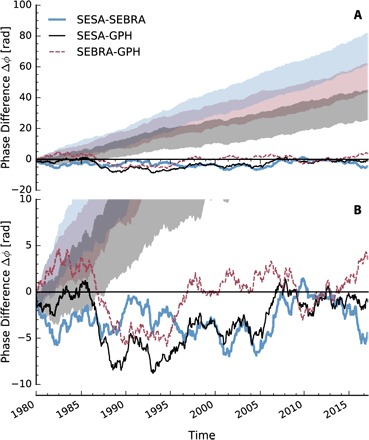
Phase difference time series. (**A**) The gray shaded area marks the 5 and 95% percentiles of phase difference time series from the 250 AR2 surrogates of the time series computed with maximum likelihood estimates of the AR2 parameters. (**B**) Zoomed in view of (A).

One way to study the phase relation of the precipitation dipole to the Rossby waves is to directly investigate the temporal evolution of the phase differences

$$\Delta \phi (t)=\phi_{i}(t)-\phi_{j}(t)$$ (3)

with the indices *i* and *j* representing either of SEBRA, SESA, and GPH. If *i* oscillates faster (slower) than *j*, then the phase differences are positive (negative). To test the statistical significance of these phase differences, we calculated 250 autoregressive surrogates of order 2 (AR2) for each of the time series. AR2 surrogates are chosen because they can oscillate with a preferred frequency [e.g., (*27*)]. The surrogates were generated on the basis of the Yule-Walker estimates of the AR2 coefficients of the unprocessed data and then processed in the same way as the actual data, including the SSA filtering. The *k*th surrogate difference is calculated as

$$\Delta \phi_{k}^{{\left(s\right)}_{k}}(t)=\frac{1}{2}((\phi_{i}(t)-\phi_{j}^{{\left(s\right)}_{k}}(t))+(\phi_{i}^{{\left(s\right)}_{k}}(t)-\phi_{j}(t)))$$ (4)

where the superscript (*s*) denotes a phase generated from the surrogates. Hence, we test whether the phase differences induced by the two observables are small against the difference induced by one of the observables and a surrogate of the other one. Similar schemes have been used to test for the statistical significance of phase coherences in previous studies [e.g., (*28*)]. The top panel of Fig. 5 shows the 5 and 95% percentiles of the 250 surrogate phase differences. Similar results can be obtained with AR1 surrogates as well (fig. S4).

By examining Fig. 5, we see that the phase differences of the data are well below the surrogates and very close to zero: The phase differences remain below 9 full periods, which is remarkable since the potential maximum of Δϕ is 350 periods, given by the number of periods the time series goes through for the complete duration of the studied interval. While it could be expected that the SEBRA-to-SESA phase difference stays close to zero, the SESA-to-GPH and SEBRA-to-GPH phases do not exhibit larger differences over the course of the dataset. More pronounced (negative) excursions of the phase differences are seen between 1985 and 1988 for SEBRA-to-GPH and SESA-to-GPH differences, indicating that GPH oscillates faster than the precipitation proxies during this time. The maximum absolute phase difference between the start and the end of a season is about 3.5, occurring during this time interval, while the mean value over all seasons is about 1.0. In addition, larger phase differences can be observed between 2005 and 2007 for the SESA-to-SEBRA and SESA-to-GPH differences, indicating a faster oscillation of the average precipitation in SESA. On average, however, the SESA-to-GPH and SEBRA-to-GPH phase differences are typically negative, indicating that the oscillations of the atmospheric waves are slightly faster than those of the precipitation dipole.

The surrogates exhibit a spectrum similar to those of the most climatic time series. However, by construction, their spectra still slightly differ from those of the investigated time series themselves, and one can see that these rather small differences lead to phase differences that are far larger than those of the three proxy time series in question to each other.

**Fig. 6:**
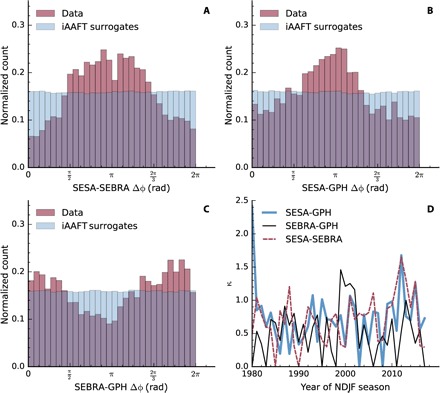
Phase difference histograms. (**A** to **C**) Histograms *H*(*x*, *y*) of phase difference of all reference time series. The empirical distributions of the observables are significantly different from iAAFT surrogates at a significance level α < 0.0001 due to a KS test. (**D**) Temporal evolution of the dispersion parameter κ of a MLE fitted von Mises distribution to the phase differences for each season.

Another possibility to study the phase relation is to directly examine the distribution of the phase differences. For this purpose, phase differences are mapped back into the interval [0,2π], and histograms *H*(*i*, *j*) of all observable pairs *i* and *j* are computed (Fig. 6). Surrogates with randomized phases [so-called iterative amplitude adjusted Fourier transform (iAAFT) surrogates (*29*); see Materials and Materials for more details], which preserve the spectrum of the original data, provide a comparison and significance test. If the phases of the observable have no relation to each other, then the histograms show a uniform distribution. A Kolmogorov-Smirnov (KS) test of the phase difference distributions against those of the iAAFT shows that the observed distributions differ from the surrogate distributions at a significance level of α < 0.0001 for all three observable pairs. While *H*(SESA, SEBRA) and *H*(SESA, GPH) exhibit phase differences in the complete interval, they both have a broad peak around π. *H*(SEBRA, GPH) displays less pronounced, but still visible peaks around 0 and 2π, respectively.

As a measure of the spread of the distribution and thus of how coherent the phases of the observables are, we perform a maximum likelihood estimation (MLE) of a von Mises distribution to the data (*30*). The von Mises distribution is an approximation to the circular wrapped normal function that exhibits an easier mathematical form than the latter. Its probability density function is *f*_vm_ (*x*) = exp (κcos (*x* − μ))/(2π*I*_0_(κ)) with the dispersion κ and location μ. *I*_0_ (κ) denotes the modified Bessel function of order zero. If the dispersion parameter κ of the von Mises distribution is zero, then the distribution is uniform, and thus, the observables are incoherent. If κ is large, the distribution resembles a normal distribution with κ^−1^ as its SD und μ as its mean. Thus, a κ significantly different from 0 and those of the iAAFT surrogates hints a phase coherence between the observables: the larger κ, the clearer. We can fit the data for each season separately and investigate the temporal evolution of the phase coherence with these time series κ_*i*,*j*_(*t*) (see Fig. 6D). While the more recent years since 2009 exhibit a strong phase coherence for all observable pairs (large κ), there are, especially for κ_SEBRA,GPH_, some seasons where the phase difference is more spread out. This occurred particularly in 1994/1995 and 1995/1996 and 2005/2006, and 2006/2007. Except for these seasons, κ_SEBRA,GPH_ boasts higher values. κ_SESA,GPH_ and κ_SESA,SEBRA_ exhibit a very similar behavior, apart from some dips for κ_SESA,GPH_ in 2003/2004 and 2008/2009.

## DISCUSSION

We have presented two complementary approaches to show how precipitation in South America, and particularly its dominant, dipolar variability mode during the monsoon season, is coupled to the southern hemisphere Rossby wave trains. This dipole is characterized by alternating wet and dry conditions between SEBRA and SESA, two of the most densely populated areas of South America.

We first showed that the spatial patterns of the two leading EOFs of the precipitation anomalies in South America can be well reproduced by a conceptual model of a traveling atmospheric pressure wave. In this conceptual model, precipitation is proportional to the spatial derivative of the wave and is hence highest at the boundary from high- to subsequent low-pressure cells in analogy with frontal systems. Composites of GPH data for times of strong precipitation in SESA and SEBRA, respectively, show concise atmospheric waves originating from the southern Pacific Ocean, which exhibit opposite phases for the two modes of the dipole. The CEOF analysis complements this approach, and the propagation of a wave along the eastern South American coast can be identified in its leading eigenmode. Together with the successful reproduction of the spatial EOF patterns, this provides strong evidence that the leading variability mode is determined by the Rossby wave activity.

To further corroborate this statement and, in particular, to quantify the dependencies caused by the corresponding mechanism, we analyzed the phase coherence between three distinct time series: two representing average precipitation in the two reference regions in SESA and SEBRA, respectively, and one representing the upper-level GPH, and hence Rossby wave activity, over southern South America. Specifically, we embedded the SSA-processed observables with a Hilbert transform and calculated the corresponding phase time series for each of them.

The observed phase differences are small for the entire time period. Given that a phenomenon at intraseasonal time scales is studied and that each observable completes roughly 350 periods in the course of the studied time span, this is a remarkable coherence. Of course, the three investigated observables cannot be expected to be in perfect phase synchronization for the entire span of the studied interval, given that many different factors, such as orographic barriers and nonlinear effects related to convection, potentially play a role.

The dominant moisture source of precipitation in subtropical South America is the southward flow from the tropics related to the South American low-level jet (*3*, *6*, *31*). The flow direction at the outlet of this jet is determined by the pressure configuration between SESA and SEBRA and, hence, by the alternation of low- and high-pressure cells caused by the Rossby waves. If a low-pressure cell is located just south of SESA, then this flow transports moisture along the isobars toward SESA and correspondingly for SEBRA. The fact that the GPH-derived Rossby wave time series tend to oscillate slightly faster than the precipitation in SESA and SEBRA can be explained along these lines: If the northward propagating pressure waves occasionally fail to pick up the moisture flow from the tropics along the isobars, then one would effectively obtain slightly faster oscillations in the driving pressure waves than in the responding precipitation waves.

In addition, positive feedbacks between moisture flow and convection related to the release of latent heat may lead to slight deviations from a perfect phase coherence. Notably, the atmospheric waves propagating northward across the South American subtropics occasionally become stationary, establishing prolonged episodes of an active SACZ of the order of 5 days (*6*, *7*, *32*).

The histograms of the phase differences exhibit visible peaks that are significantly different from those of phase-randomized surrogates. To make a justified assertion about the phase coherence of the precipitation dipole and Rossby wave, we need both the fact that their phase differences stay close to zero for the whole duration of the study period and that their distributions exhibit distinguished peaks.

Since we included most of the intraseasonal SSA components in our analysis, our results show that the interaction of the precipitation dipole with the Rossby waves is one of the dominant factors of intraseasonal precipitation variability in South America: Our results indicate that the dipole-like pattern is not present because of some direct interaction between the climatic subsystems in SESA and SEBRA but is rather caused by the propagation of Rossby wave trains from the southern Pacific Ocean, along the southern tip of the South American continent, and then northward toward the subtropical Atlantic Ocean. The identification of this causal mechanism, which explains the dominant variability mode of monsoonal precipitation in South America, should help to improve the synoptic-scale predictability of precipitation particularly in SESA and SEBRA, which are the two regions that are most affected by this mode. The presented framework also enables us to investigate the temporal evolution of phase coherence at longer time scales and, hence, the interannual variability of the South American precipitation dipole. Seasons with larger phase difference should be investigated for dependencies with other variabilities such as the ENSO or the MJO in future work.

## MATERIALS AND METHODS

### Data

For this study, precipitation and GPH data at 250 hPa from NASA’s Modern-Era Retrospective Analysis for Research and Applications, version 2 (MERRA2) were used (*33*). The dataset covers the period from 1980 to 2016 and consists of 6-hourly data on a 1/3° × 2/3° rectangular grid. The precipitation data were smoothed using a moving average with a window size of 4 days. The two reference regions for the precipitation dipole were chosen in accordance with previous research (*12*), and the mean of all grid cells within these boxes was used as an index for the precipitation in SEBRA and SESA, respectively. The reference region for inferring Rossby wave activity was chosen over southern South America (see Fig. 1). The results we report below are very robust to changes in position and size of this box; it is important, however, that the reference region is not much larger than half of a typical wavelength of the wave train to still properly capture its oscillating behavior. The mean of the GPH at 250 hPa of all grid nodes within this reference thus serves as an index for the Rossby wave train. Figure 1 (A and B) shows the reference regions and the typical opposing configuration of the Rossby wave trains in the GPH anomaly fields during extreme precipitation (above the 90th percentile) in SEBRA and SESA. The precipitation itself is shown in Fig. 1 (C and D). The South American precipitation dipole is a phenomenon restricted to the austral summer from NDJF. Because some of the methods used here are easier to handle with data that have a regular time axis without jumps, all-year data were used at first. The crucial parts of the analysis are, however, limited to the NDJF data.

### Conceptual model

A key hypothesis we intend to test in this study is that the variability mode corresponding to the South American precipitation dipole could be explained by northward propagating waves triggered by the southern hemisphere Rossby wave train. For this purpose, we first introduced the following conceptual model: Denoting the dimensionless GPH along an arbitrary direction *x* as *h* and the precipitation along the direction as *p*, we chose to model *h* with a wave equation

□h(x,t)=0 (5)

with the one-dimensional d’Alembertian $□=\frac{1}{c}\frac{\partial^{2}}{{\partial t}^{2}}-\frac{\partial^{2}}{{\partial x}^{2}}$ and *p* as its spatial derivative

$$p(x,t)=\frac{\partial }{\partial x}h(x,t)$$ (6)

since precipitation, on a large scale, typically occurs at the fronts between highs and subsequent lows. This equation solves to a traveling wave for *p*(*x*, *t*). By embedding this traveling wave in the same grid as the data and adding Gaussian damping along and perpendicular to the propagation direction, we generated the model data *P*_M_(λ, ϕ, *t*). Its parameters are the mean values of the Gaussian damping λ_0_, ϕ_0_, their SDs σ_λ_, σ_ϕ_, the wavelength *L*, and the direction θ of the wave (see the Supplementary Materials for the full equations). The model data *P*_M_(λ, ϕ, *t*) could be used to calculate the first two EOFs of the conceptual model. These EOFs were then fitted, by optimizing the model parameters via least squares, to the EOFs of the precipitation data (see Fig. 2). While the parameters referring to the Gaussian damping and the direction θ roughly account for the location and orography, the wavelength or wave number is an important parameter of the modeled wave.

The CEOF analysis extends the standard EOF analysis by applying the PCA to the complexified time series, i.e., the analytical signal [e.g., (*21*)]. The analytical signal $\tilde{x}(t)$ is usually computed by augmenting the time series with its Hilbert transform as its imaginary part, so that $\tilde{x}(t)=x(t)+iH(x(t))$. The Hilbert transform H(f(x)) is defined as

$$H(f(x))=\frac{1}{\pi }P.V.\int_{-\infty }^{+\infty }\frac{f(\tau )}{x-t}d\tau$$ (7)

with P.V. denoting the Cauchy principal value of the integral. It induces a 90° phase shift to every frequency component of the time series. Figure 4 shows an example of a Hilbert transform and the signal it was calculated from. This two-dimensional embedding of the time series enabled us to analyze oscillations in time series with methods that rely on phase information, as we have used here throughout the article. Hence, the CEOF method is especially well suited for identifying oscillatory patterns and propagating waves (*20*). We followed here the notation of Barnett (*20*): The eigenvectors *B*_*n*_(**x**) of the covariance matrix of the spatiotemporal complexified data $\tilde{X}(x,t)$ and its principal components $A_{n}(t)=\sum_{x}\tilde{X}(x,t)B_{n}^{*}(x)$ are all complex valued and can therefore not be analyzed directly, as it is the case for the standard EOF analysis. Thus, we investigated the following three measures, which separate the temporal and spatial domain, as well as the phase and amplitude information:

1) Spatial phase function $\theta_{n}(x)=\;\text{arctan}(\frac{ℑ\{B_{n}(x)\}}{ℜ\{B_{n}(x)\}})$

2) Spatial amplitude function $S_{n}(x)={\left(B_{n}(x)B_{n}^{*}(x)\right)}^{1/2}$

3) Temporal phase function $\phi_{n}(t)=\;\text{arctan}(\frac{ℑ\{A_{n}(t)\}}{ℜ\{A_{n}(t)\}})$

More details on CEOF analysis are given in (*20*).

### Singular spectrum analysis

The precipitation dipole is an intraseasonal phenomenon. To remove the annual cycle as well as high-frequency oscillations and noise, we applied SSA (*17*, *18*). SSA has been successfully applied to investigate intraseasonal climate phenomena before [e.g., (*34*). Similar to PCA, but focusing on the temporal rather than the spatial domain, SSA solves an eigenvalue problem and decomposes a single time series into several components that can be ordered by the amount of variance of the time series they account for. To accomplish this, first, the time series *x*(*t*), which here denotes either of the three observables, was delay embedded into a τ × *N* matrix **X** with the *k*th row given by *x*(*t* + *k*), the time series delayed by *k*. Thereafter, the eigenvalue problem of the covariance matrix of **X** was solved. It can be shown that the magnitude of the eigenvalues is directly proportional to the amount of variance that is accounted for by the respective eigenvectors. With these eigenvectors, we could also reconstruct different parts of the original time series, corresponding to the eigenvalues one is interested in. This allows, e.g., to filter out certain variability modes or frequency bands from a given time series. Subsequently, we investigated these reconstructed SSA components for each of the three time series under study. The reconstructed components have been shown to capture the phase of the time series well (*35*), which is a necessary condition for our investigations. This left us with the task of selecting the right components for our investigation.

### Phase reconstruction

Similar to two chaotic oscillators that begin to synchronize once brought into contact with each other [e.g., (*15*)], we also expected oscillatory climatic subsystems that are coupled to each other to exhibit this behavior. To infer the phase coherence between two observables, we first needed a two-dimensional embedding of each time series. A common approach for this purpose is to calculate the analytic signal of the time series via a Hilbert transform (*15*), which is defined in Eq. 7. To define a meaningful phase of the time series, this signal needs to exhibit a well-centered oscillation around a common reference point. Instead of the time series itself, Osipov *et al*. (*36*) argue that it is also possible to define a meaningful phase by using the derivative and its Hilbert transform. This results in a more concise definition of the phase, since the derivative is better centered than the time series itself, and slow variations are eliminated (*16*). The derivatives were calculated with the standard fourth-order finite differences formulas. Thus, denoting *x*(*t*) as any of the three time series, we defined its phase as

$$\phi (t)=\;\text{arctan}\frac{H\{\dot{x}\}(t)}{\dot{x}(t)}$$ (8)

Figure 4 shows an example of a time series and its embedding. We saw that the definition of the phase in the above described way is justified, since most oscillations revolve around the origin. After each full period, 2π was added to unwrap the phase. As we were investigating a seasonal phenomenon, we were interested only in phase coherence during the NDJF season. Thus, we only considered NDJF data and performed an end point matching to concatenate the data of different seasons. The end point matching minimizes the Euclidean distance between the joint vector of all three time series, their derivatives and Hilbert transforms, as well as an additional penalty that is linear in time, and favors end points late in the season and start points early in the season.

Aside from investigating and comparing the phase difference time series, we also investigated the histogram of the phases of all observables (see Fig. 6). To assess the significance of these phase histograms, we used iAAFT surrogates. These surrogates are refined Fourier transform surrogates. Fourier transform surrogates were computed by multiplying the Fourier-transformed time series with a random phase vector and transforming it back into the original space. Therefore, the surrogates exhibit the same spectrum as the original time series but have randomized phases. For a detailed account of these surrogates, see (*29*).

## Supplementary Material

http://advances.sciencemag.org/cgi/content/full/4/12/eaau3191/DC1

## SUPPLEMENTARY MATERIALS

Supplementary material for this article is available at http://advances.sciencemag.org/cgi/content/full/4/12/eaau3191/DC1 Fig. S1. Eigenvalue spectrum of the PCA performed with precipitation anomalies from MERRA2 shown in Fig. 2. Fig. S2. Spatial phase of the first CEOF of the conceptual model. Fig. S3. SSA of all three investigated observables. Fig. S4. Phase difference time series results analogous to Fig. 5. Fig. S5. Phase difference time series results analogous to Fig. 5. Fig. S6. Phase difference histogram results analogous to Fig. 6. Fig. S7. Phase difference time series results analogous to Fig. 5. Fig. S8. Phase difference histogram results analogous to Fig. 6.
