# Supplementary material for: Phase coherence between precipitation in South America and Rossby waves
Source: Sci Adv. 2018 Dec 19;4(12):eaau3191. doi: 10.1126/sciadv.aau3191 (PMC6300402; doi:10.1126/sciadv.aau3191)
Supplement: http://advances.sciencemag.org/cgi/content/full/4/12/eaau3191/DC1 [file supp_4_12_eaau3191__index.html]

Science Advances | Science Advances

## Supplementary Materials

**This PDF file includes:**

- Fig. S1. Eigenvalue spectrum of the PCA performed with precipitation anomalies from MERRA2 shown in Fig. 2.
- Fig. S2. Spatial phase of the first CEOF of the conceptual model.
- Fig. S3. SSA of all three investigated observables.
- Fig. S4. Phase difference time series results analogous to Fig. 5.
- Fig. S5. Phase difference time series results analogous to Fig. 5.
- Fig. S6. Phase difference histogram results analogous to Fig. 6.
- Fig. S7. Phase difference time series results analogous to Fig. 5.
- Fig. S8. Phase difference histogram results analogous to Fig. 6.

Download PDF

**Files in this Data Supplement:**

- Adobe PDF - aau3191\_SM.pdf
