# Supplementary material for: Phase coherence between precipitation in South America and Rossby waves
Source: Sci Adv. 2018 Dec 19;4(12):eaau3191. doi: 10.1126/sciadv.aau3191 (PMC6300402; doi:10.1126/sciadv.aau3191)
Supplement: http://advances.sciencemag.org/cgi/content/full/4/12/eaau3191/DC1 [file aau3191_SM.pdf]

Science Advances
AAAS

advances.sciencemag.org/cgi/content/full/4/12/eaau3191/DC1

Supplementary Materials for

Phase coherence between precipitation in South America and Rossby waves

Maximilian Gelbrecht*, Niklas Boers, Jürgen Kurths

*Corresponding author. Email: gelbrecht@pik-potsdam.de

Published 19 December 2018, *Sci. Adv.* **4**, eaau3191 (2018)
DOI: 10.1126/sciadv.aau3191

This PDF file includes:

Fig. S1. Eigenvalue spectrum of the PCA performed with precipitation anomalies from MERRA2 shown in Fig. 2.
Fig. S2. Spatial phase of the first CEOF of the conceptual model.
Fig. S3. SSA of all three investigated observables.
Fig. S4. Phase difference time series results analogous to Fig. 5.
Fig. S5. Phase difference time series results analogous to Fig. 5.
Fig. S6. Phase difference histogram results analogous to Fig. 6.
Fig. S7. Phase difference time series results analogous to Fig. 5.
Fig. S8. Phase difference histogram results analogous to Fig. 6.

Conceptual Model

The full equations of the conceptual model are represented using the centered coordinates

$$\xi = \varphi - \varphi_0 \tag{1}$$

$$\nu = \lambda - \lambda_0 \tag{2}$$

and the coefficients

$$a = \frac{\cos^2\theta}{2\sigma_\xi^2} + \frac{\sin^2\theta}{2\sigma_\nu^2} \tag{3}$$

$$b = -\frac{\sin 2\theta}{4\sigma_\xi^2} + \frac{\sin 2\theta}{4\sigma_\nu^2} \tag{4}$$

$$c = \frac{\sin^2\theta}{2\sigma_\xi^2} + \frac{\cos^2\theta}{2\sigma_\nu^2} \tag{5}$$

that use the propagation direction θ and the standard deviations σ_ξ and σ_ν in the model data equation

$$P_M\left(\xi(\varphi), \nu(\lambda), t\right) = \exp\left(-\left(a\xi^2 + 2b\xi\nu + c\nu^2\right)\right)\sin\left(\left(2\pi\left(\cos\theta\cdot\xi + \sin\theta\cdot\nu\right)\right)/L - \omega t\right) \tag{6}$$

(7)

While the wavelength L is another parameter of the model, the (temporal) frequency is kept at $\omega = 1/40\,\mathrm{d}$ as it does not affect the spatial structure of the resulting EOFs. The full set of parameters is thus $(\varphi_0, \lambda_0, K, \sigma_\xi, \sigma_\nu, \theta)$. We then calculate the model $\mathrm{EOF}_i^{(M)}$ and fit it to the data by numerically minimizing the least square error to the observed data $\mathrm{EOF}_i^{(D)}$

$$\mathrm{LSE}\left(\varphi_0, \lambda_0, , \sigma_\xi, \sigma_\nu, \theta\right) = \sum_{\varphi,\lambda}\sum_{i=1}^{2}\left(\mathrm{EOF}_i^{(M)}(\varphi,\lambda) - \mathrm{EOF}_i^{(D)}(\varphi,\lambda)\right) \tag{8}$$

As a single function evaluation can take a few seconds on a current processor, this is obviously a computationally relative expensive minimization that does very likely exhibit multiple local minima. We used the Nelder-Mead implementation of Python's scipy with initial parameter guesses chosen by hand to be already relatively close to being a good fit.

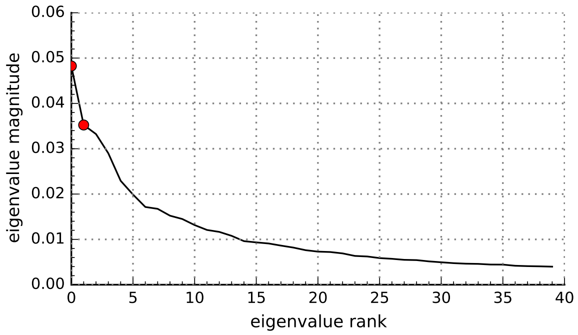

Fig. S1. Eigenvalue spectrum of the PCA performed with precipitation anomlies from MERRA2 shown in Fig. 2. The red circles indicate the two leading EOFs that are further investigated with the conceptual model.

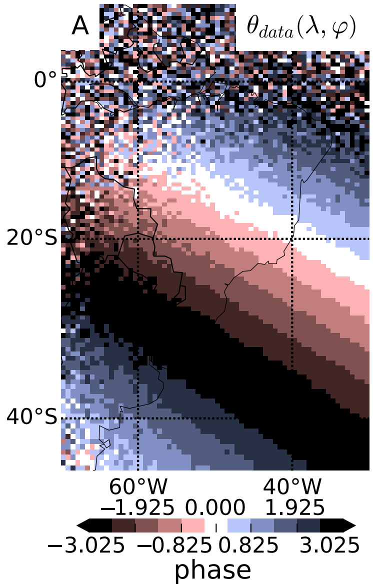

Fig. S2. Spatial phase of the first COEF of the conceptual model.

Monte-Carlo SSA

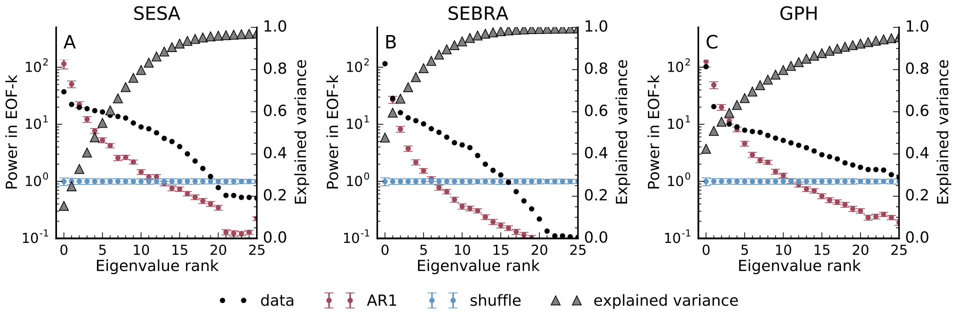

Fig. S3. SSA of all three investigated observables. 1000 AR1 and shuffle surrogates are generated for each of the observables and can serve as significance tests for the SSA components.

Addditonal results with AR1 surrogates

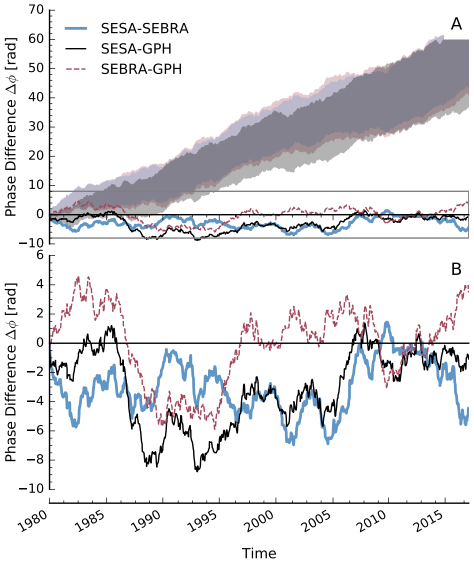

Fig. S4. Phase difference time series results analogous to Fig. 5. These results feature AR1 surrogates instead of the AR2 surrogates presented in the main text.

Additional results with alternative preprocessing methods

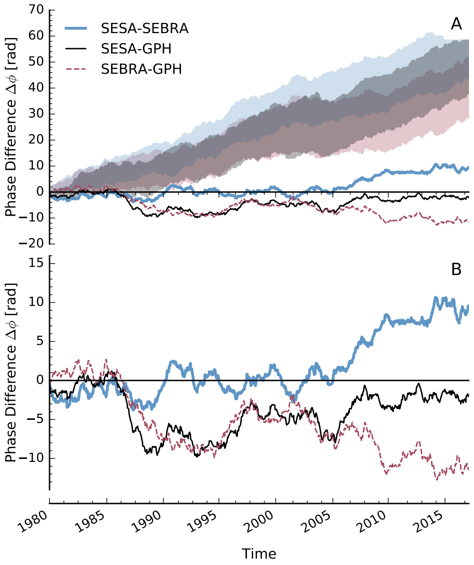

Fig. S5. Phase difference time series results analogous to Fig. 5. These results were computed with the third and fourth SSA component included in the analysis

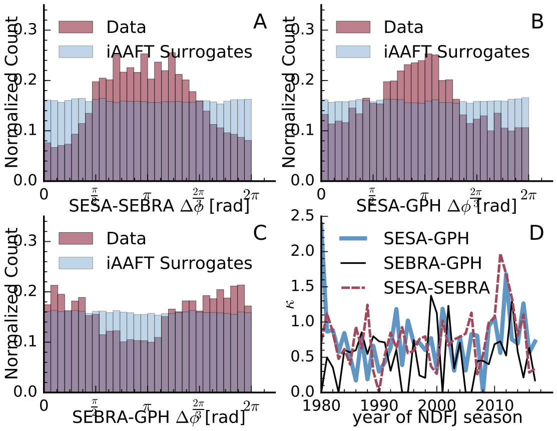

Fig. S6. Phase difference histogram results analogous to Fig. 6. These results were computed with the third and fourth SSA component included in the analysis.

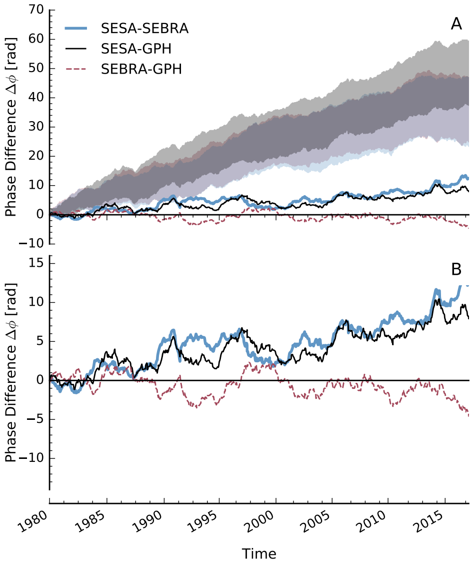

Fig. S7. Phase difference time series results analogous to Fig. 5. These results were computed with a 10-50 day Bandpass-Lanczos Filter with 1501 weights instead of the SSA.

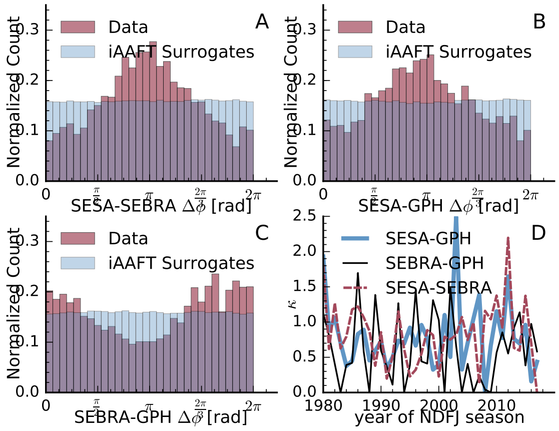

Fig. S8. Phase difference histogram results analogous to Fig. 6. These results were computed with a 10-50 day Bandpass-Lanczos Filter with 1501 weights instead of the SSA.
